# Current considerations concerning endodontically treated teeth: alteration of hard dental tissues and biomechanical properties following endodontic therapy

**Published:** 2009

**Authors:** Bogdan Dimitriu, Constantin Vârlan, Ioana Suciu, Virginia Vârlan, Dana Bodnar

**Affiliations:** * “Carol Davila” University of Medicine and Pharmacy Bucharest, Faculty of Dental Medicine, Department of Endodontics; ** “Carol Davila” University of Medicine and Pharmacy Bucharest, Faculty of Dental Medicine, Department of Operative Dentistry

**Keywords:** Endodontically treated teeth, biomechanical properties, dentin physico-chemical characteristics

## Abstract

The aim of this general article is to present an overview of the current knowledge about composition and structural changes and also about specific biomechanical alterations related to vitality loss or endodontic therapy. For a long time, these issues have been controversially approached from a clinical standpoint and are therefore still confusing for many practitioners.

Vitality loss or endodontic procedures seem to induce only negligible changes in hard dental tissue moisture. Physico-chemical properties of dentin can be modified by some of the endodontic chemical products used for chemo-mechanical debridement. On the other hand, tooth biomechanical behavior is affected, since tooth strength is reduced proportionally to coronal tissue loss, due to either pre-existent carious/non-carious lesions or cavity acces preparation, besides restorative procedures.

The related literature shows the lack of accepted clinical standards and consensus regarding the optimal way of approaching the specific tooth biomechanics following endodontic therapy.

## Introduction

The ultimate goal of endodontic treatment consists of maintaining a functional pulpless tooth by means of adequate restoration. The initial clinical situation is the main aspect to be taken into consideration for achieving a succesful result.

Same as in other medical specialities, a single general algorithm able to solve any of the very specific clinical situations involving restoration of endodontically treated teeth, does not exist. Moreover, the dentist has to deal with the risk management of every distinct case.

In order to be restored, endodontically treated teeth often require a special approach, due to the considerable loss of tooth structure, the existing carious or non-carious coronal lesions, previous restorations and the endodontic treatment itself. On the other hand, the diminished resistance to functional occlusal forces of the remaining hard dental tissues is an obvious clinical observation. Some aspects concerning the etiology of this situation are still controversial.

## Hard dental tissue composition and physical characteristics 

A change in the physical properties of the remaining hard dental tissues is assumed by many dentists, but there is no indisputable evidence to sustain this otherwise widespread opinion. There are structural differences of the dentinal collagen: more incomplete bindings can be found into the collagen of non-vital teeth compared to the vital ones. The weakening of collagen network induced by dentinal dehydration was also taken into account. In a normal, healthy, vital tooth, the dentinal fluid flow is under a slight positive pressure of 15 cm H2O (1,47 kPa) [**1**,**2**,**3**]. The pressure is thought to be present due to the blood flow through the pulpal tissues, which is by far excessive compared to the amount required to nourish the pulpal tissues [**4**]. What happens to the dentin when that fluid flow is altered by blood supply removal, remains an issue that needs to be further thoroughly discussed about. 

The change in tooth moisture content due to loss of vitality [**5**,**6**] has a slight influence on Young modulus [**7**]. This change in water content has no influence in decreasing compressive and tensile strength [**7**]. It is the change in free water and not in the bonded one that accounts for the 9% loss of moisture [**5**].

For many years, it was thought that the physical properties could be expressed for dentin and enamel as if the structures were isotropic. Acknowledged classical research (Craig and Peyton – 1958) measured the elastic and mechanical properties of dentin. It was stated that the modulus of elasticity was independent of the orientation of the samples and its average was between 2,4-2,7 x 106 psi [**8**]. This is equivalent to 16,55-18,62 GPa, that is the measurement function used in modern studies. In a more recent research of the elastic modulus which describes the brittle behavior of a substance, a 21,8 GPa value has been found in horizontally sectioned specimens - i.e., the dentinal tubules are in cross-section - versus 18,5 GPa for vertically sectioned specimens - i.e., with the dentinal tubules running parallel to the wall of the sectioned specimen [**9**].

While reviewing previous research, it was discovered that the storage medium of the tooth specimens tested for mechanical properties has significantly affected the measurements that have been taken. This finding is significant because it questions the outcome of so much of the previous research. Habelitz et al. discovered in 2002 that storing samples for testing in deionized water or CaCl2 solution, dramatically lowered the elastic modulus and the hardness of the samples because it altered their calcification. Storage in a medium of Hank’s Balanced Salt Solution (HBSS - contains various amounts of KCl, KH2PO4, NaCl, NaHCO3, Na2HPO4, CaCl2, MgCl2, and D-Glucose, and may contain phenol red) for a period of up to two weeks showed no alteration in the outcomes, whereas storage in the other mediums lowered the mechanical properties to 50% in two weeks [**10**]. This is significant because the testing methods affected the samples so much that they no longer behaved as “vital” teeth did.

While reviewing the mechanical properties of human dentin as reported in literature over the last fifty-plus years, Kinney et al. explained in 2003 how the different values have been found, and reinterpreted the data to conclude that Young’s modulus of elasticity for dentin lies between 18-25 GPa [**11**]. The study went on describing that the higher value of the elastic modulus, compared to previous research, was evaluated by a micromechanical calculation model rather than a bounding mathematical model which was previously used. It stated that dentin is viscoelastic, i.e. the dentin exhibits a time-dependent response to stress and its components are anisotropic. Other researchers concur with this finding [**12**,**13**]. Young’s modulus was defined as minimal in the direction of the dentinal tubules and monotonically increased to maximum at the plane of the mineralized collagen fibrils. The collagen fibrils run perpendicular to the structure of the dentinal tubules. Normal dentinal tubules generally run perpendicular to the external surface of the tooth. It was noted that, due to several methods which the modulus is measured by, different values are reported. The study found that creep relaxation, due to the viscoelastic behavior of dentin, explains the differences in elastic modulus values obtained between resonant ultrasound spectroscopy and the atomic force microscope (AFM) using nanoindentation methods [**10**,**14**]. The benefit of the AFM method is that it can separately measure the various components of dentin or enamel in a wet environment, which is essential to the reporting of correct values, as dehydrated dentin has a higher elastic modulus and is, therefore, more brittle [**14**]. This is significantly greater than values previously reported (13-16GPa) and is consistent with values reported for bone.

## Physico-chemical properties of dentin

Studies of tissue composition found no difference in collagen cross linkage between vital and non-vital dentin [**15**]. The removal of pulpal tissue is not responsible for any chemical alteration of dentin. On the other hand, some of the endodontic chemical products used for chemo-mechanical debridement, which are hypochlorite, chelators and calcium hydroxide, can interact with the root canal of the dentin and modify its characteristics.

The use of sodium hypochlorite as a root canal irrigating solution is due mainly to its efficacy for pulpal dissolution and antimicrobial activity. Low concentration of sodium hypochlorite solution, such as 1%, presents acceptable biocompatibility. A higher concentration which is also used in endodontic therapy and having the value up to 5,25%, of sodium hypochlorite has clearly proved the effect of softening the dentine, if the action time exceeds 10 minutes.

**Fig. 1:**
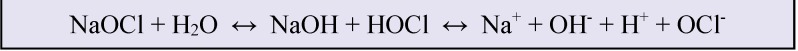
Dynamic balance of sodium hypochlorite

Sodium hypochlorite exhibits a dynamic balance as it is shown in **Fig. 1**.

**Fig. 2:**
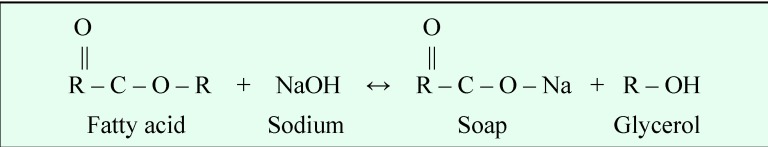
Saponification reaction

**Fig. 3:**
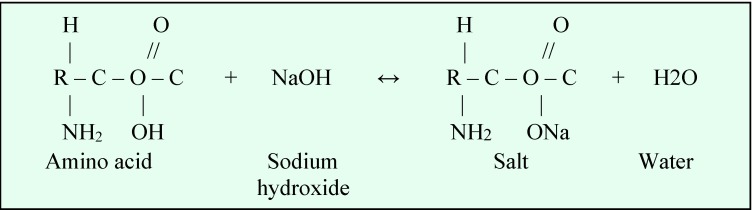
Amino acid neutralization reaction

**Fig. 4:**
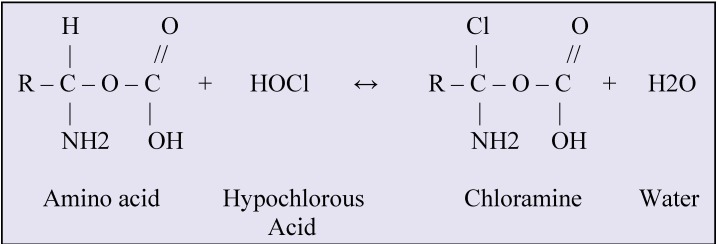
Chloramination reaction

The chemical reactions between organic tissue and sodium hypochlorite are shown in **Fig. 2**-**4**.

Considering the physico-chemical properties of sodium hypochlorite when in contact with organic tissue, these reactions can be verified. Sodium hypochlorite is a strong base (pH>11). At 1% concentration, sodium hypochlorite presents a surface tension equal to 75 dynes/cm, stickiness equal to 0.986 cP, 65.5 mS of conductivity, 1.04 g/cm3 of density and moistening capacity equal to 1 h and 27 min. [**16**].

Sodium hypochlorite alters the organic substrate of dentin [**17**,**18**] and exhibits a proteolytic action. Depletion of the organic phase was confirmed by infrared spectroscopy [**19**]. This is assigned to extensive fragmentation of long peptide chains, including collagen [**20**], leading to a reduced modulus of elasticity and flexural strength of dentine [**21**].

Chelators such as ethylene-diaminetetra-acetic acid (EDTA), 1,2 cyclohexane-diaminetetra-acetic acid (CDTA), and ethylene-glycol-ether diaminetetra-acetic acid (EGTA), as well as calcium hydroxide, widespread used for canal irrigation and disinfection, interact with the mineral content of dentin. The result is dentin erosion and softening, since they mainly deplete calcium through complex formation and also affect non-collagenous proteins: proteoglycans, dentin phosphoproteins and sialoproteins. [**17**,**22**,**23**,**24**].

## Tooth biomechanics and fracture resistance

The specific biomechanical behaviour of endodontically treated teeth is first of all induced by the loss of hard dental tissues because of carious and non-carious lesions, which may lead to pre-existent cavity preparation, before endodontic therapy, besides the dimensions of the cavity acces. Removing the pulp chamber roof, with consequent deepening of the whole cavity will increase flexure possibilities of the vertical coronal walls, which become more prone to fracture [**25**].

A conservative endodontic cavity access preparation will affect tooth stiffness with only 5%. The subsequent root canal therapy (instrumentation and obturation) will lead to a slight reduction in fracture resistance [**26**] or will have little or no effect on biomechanical properties of the tooth [**27**].

Cleaning and shaping the root canal system diminishes tooth stiffness proportional to the amount of removed tissue and it is possibly related to the chemical or structural alteration brought about by endodontic chemical products [**28**,**29**,**30**,**31**].

The highest risk of tooth fracture is caused by additional MO, DO and especially MOD cavity preparations, which means loss of marginal ridges. Tooth stiffness reduction is reported to be 14% to 44% and 20% to 63%, respectively [**27**,**32**,**33**]. Additional research on the influence of residual hard dental tissue on stiffness and deformation under stress of endodontically treated teeth, revealed that maximum tooth fragility results from an endodontic cavity access combined with a MOD preparation [**34**,**35**]. The cavity configuration, its depth and isthmus width are extremely critical factors with respect to fracture risk [**34**,**35**,**36**,**37**].

Increased tooth resistance to fracture can be achieved by a larger amount of residual hard dental tissue[**38**,**39**] and by preserving and maintaining cervical tissue to create the ferrule effect [**40**]. In order to stabilize the restored endodontically treated tooth, a minimal 1mm ferrule is considered to be necessary [**38**].

Despite lack of clear evidence, absence of the dental pulp may subsequently lead to loss of some of the mechanical responsiveness of the tooth, diminishing its proprioceptive sensitivity [**25**].

Although specialized proprioceptors could not be identified in the dental pulp, there is evidence of A-β nerve fibres presence which have well documented proprioceptive functions. Therefore, the modified sense perception of occlusal forces in non-vital teeth may produce discomfort only when reaching the double values of the functional occlusal load for the vital teeth [**25**].

Together with the preparation of an access cavity, canal enlargement during endodontic procedures and use of specific chemicals, all of which significantly reduce tooth strength, the above mentioned concept may offer an explanation for the higher rate of mechanical failure of endodontically treated teeth, compared to the vital ones.

## Conclusions

The loss of vitality impact appears to be moderate to negligible with concern to loss of moisture or physical properties of dentin such as microhardness, modulus of elasticity and fracture resistance.

The preparation of cavity access, canal enlargement during endodontic procedures and use of specific chemicals, however, significantly reduce tooth strength. In fact, the tissue conservation is the most critical issue when dealing with a nonvital tooth. Preserving and maintaining intact structures throughout the tooth are especially crucial to optimize the biomechanical behavior and to increase the fracture resistance of the restored endodontically treated tooth.
